# Concurrent, Convergent, and Discriminant Validity of the *DSM-5* Section III Psychopathy Specifier

**DOI:** 10.1177/10731911221124344

**Published:** 2022-09-19

**Authors:** Erin K. Fuller, Dylan T. Gatner, Kevin S. Douglas

**Affiliations:** 1 Simon Fraser University, Burnaby, British Columbia, Canada; 2 British Columbia Mental Health and Substance Use Services, Vancouver, Canada; 3 Helse Bergen Sikkerhet Kompetansesenter, Norway; 4 Oslo University Hospital, Norway

**Keywords:** psychopathy, antisocial personality disorder, psychopathy specifier, *DSM-5* alternative model for personality disorders, boldness, triarchic model of psychopathy

## Abstract

Section III of the fifth iteration of the *Diagnostic and Statistical Manual of Mental Disorders* (*DSM*-5) includes an alternative model of personality disorder diagnosis that conceptualizes antisocial personality disorder as an interpersonal, rather than behavioral, construct. However, the diagnostic specifier for psychopathy has been met with recent controversy due to its conceptual and empirical overlap with triarchic boldness, which has been debated as a necessary and sufficient domain of psychopathy. This study examined the concurrent, convergent, and discriminant validity of the specifier using canonical correlation analysis in samples of undergraduate students (*N* = 224) and community adults with prior criminal involvement (*N* = 306). Findings highlight the specifier as a multidimensional construct with divergent associations across its three facets. There was limited validity evidence for two of the three facets, raising concerns regarding the clinical utility of the psychopathy specifier.

Despite decades of empirical research, the construct of psychopathic personality disorder (or, psychopathy) has been widely disputed, in terms of both its operationalization and measurement. Most accepted definitions regard psychopathy as a personality disorder with core deficits across interpersonal (e.g., manipulativeness, grandiosity), affective (e.g., callousness, lack of remorse), and behavioral (e.g., impulsivity, irresponsibility) domains (Cooke et al., 2001; Cooke & Logan, 2015; Crego & Widiger, 2015; Hart & Cook, 2012). However, not all scholars concur on how such deficits manifest and multiple models have been posited in an effort to address growing conceptual and measurement concerns. One such model is the triarchic model of psychopathy (Patrick et al., 2009), which asserts there are three distinct psychopathy domains: boldness, meanness, and disinhibition. Meanness (i.e., callousness/cold-heartedness) and disinhibition (i.e., impulsivity/irresponsibility) have been empirically and conceptually linked to other operationalizations psychopathy and to violence (Drislane et al., 2014; Miller et al., 2016). However, conceptual and empirical investigations of boldness have remained equivocal and contentious (Marcus et al., 2013; Miller & Lynam, 2012).

Boldness is defined as social assertiveness, emotional resilience, and fearlessness (Patrick et al., 2009) and has been likened to the fearless-dominance facet of the Psychopathic Personality Inventory–Revised (PPI-R; Lilienfeld & Widows, 2005) and the emotional stability facet from the Elemental Psychopathy Assessment (EPA; Lynam et al., 2011; see Sellbom & Phillips, 2013). From a trait perspective, it has generally been regarded that the aforementioned constructs (i.e., Triarchic Psychopathy Measure [TriPM] boldness, PPI-R fearless-dominance, and EPA emotional stability) represent low-neuroticism/high-extraversion traits (Blagov et al., 2015; Crego & Widiger, 2016; Donnellan & Burt, 2016; Poy et al., 2014). Hereafter, the term “boldness” will refer to such low-neuroticism/high-extraversion traits.

## The Boldness Controversy

The construct of boldness has been theoretically controversial due to its limited convergent associations (*r* = .06–.39) with other psychopathy measures (Drislane et al., 2014; Marcus et al., 2013; Miller et al., 2016; Miller & Lynam, 2012; Sleep et al., 2019), nonexistent to weak meta-analytic associations with outcomes linked to psychopathy—for example, general criminality and violence (Marcus et al., 2013; Sleep et al., 2019), and poor incremental predictive validity (above and beyond meanness and disinhibition) for antisocial outcomes (Gatner et al., 2016; Hanniball et al., 2019). In addition, recent empirical work has suggested that boldness is typically unrelated or inversely related to generally unhealthy (or, dysfunctional) outcomes (e.g., impulsivity, risky behaviors; Anestis et al., 2019; Donnellan & Burt, 2016; Gatner et al., 2016; Hanniball et al., 2019). Moreover, prototypicality (e.g., Miller et al., 2016) and lexical similarity (Gatner et al., 2018) studies have found weak support for boldness’ inclusion in prototypical symptoms and lexical descriptions of psychopathy. Additionally, meta-analytic findings (Miller & Lynam, 2012; Sleep et al., 2019) and other empirical findings (Blagov et al., 2015; Bleidorn et al., 2019; Drislane et al., 2014; Hanniball et al., 2019; Latzman et al., 2018) have suggested strong associations between boldness and happiness, psychological adjustment, self-esteem, self-regulation, and stress immunity—variables with some support as protective factors for outcomes associated with psychopathy (e.g., violence). Such findings have generated criticisms of the viability of boldness as a necessary and sufficient component in the operationalization of psychopathy (e.g., Miller & Lynam, 2012).

Despite such criticisms, other researchers (e.g., Lilienfeld et al., 2012) have argued that boldness is a central and necessary component of psychopathy, as it is reflective of their interpretations of Cleckleyan’s (1941, 1976) descriptions that low levels of anxiety and fearlessness are predominant traits in the disorder (see Patrick, 2006, 2018, for a review). In contrast to some of the aforementioned research, other meta-analytic works have found moderate associations (*r* = .39–.44) between boldness and non-Psychopathy Checklist (PCL)–based measures of psychopathy (Lilienfeld et al., 2016). Recent empirical studies have found evidence of incremental validity of boldness in the prediction of Psychopathy Checklist–Revised (PCL-R) psychopathy (Murphy et al., 2016; Venables et al., 2017; Wall et al., 2015) and recent prototypicality studies found support for the inclusion of boldness (e.g., Berg et al., 2017; Sörman et al., 2016). In contrast to findings demonstrating null or inverse associations with constructs related to psychopathy, other studies have found positive associations with outcomes such as school misconduct, sensation-seeking, substance use, and general antisociality (Blagov et al., 2015; Coffey et al., 2018; Drislane et al., 2014, 2018; Gray et al., 2019; Marcus et al., 2013; Miller & Lynam, 2012; Poythress & Hall, 2011). Relatedly, some scholars also posit that the “adaptive features associated with psychopathy” may “mask” functional impairment (Berg et al., 2017, p. 3; Lilienfeld et al., 2016; McKinley et al., 2018).

Notwithstanding some evidence in support of boldness’ role in the disorder, psychopathy, by definition, ought to be associated with some level of functional impairment, dysfunction, or distress either of inward or of outward focus to be considered a disorder (American Psychiatric Association [APA], 2013; Livesley & Jang, 2000). Thus, an investigation of adaptive features appears counterintuitive, as it is generally understood that a personality disorder reflects impaired adaptive personality functioning (Livesley & Jang, 2000). Any association between boldness and adaptive outcomes raises significant construct validity concerns (i.e., measures of boldness may be capturing personality traits of self-assuredness and self-efficacy that are wholly unrelated to psychopathy).

## ***DSM-5*** and Psychopathy

The fifth iteration of the *Diagnostic and Statistical Manual of Mental Disorders* (*DSM*-*5*; APA, 2013) included an alternative model for personality disorders (AMPD) in Section III (i.e., *Emerging Measures and Models*). The alternative model emphasizes impairment in self and interpersonal functioning (Criterion A) and presents a dimensional trait model of pathological personality that manifests on a continuum (Criterion B; APA, 2013).^1^ The model consists of five broad domains and 25 specific traits, which are considered maladaptive derivations of the five-factor model (FFM) of personality (APA, 2013) and are measured using the Personality Inventory for *DSM-5* (PID-5; Krueger et al., 2012). Personality traits are considered in a matter of degree and are operationalized as stable dispositional tendencies to think, feel, perceive, or behave in a consistent manner across various contexts (APA, 2013).

In this alternative model, psychopathy is used as a specifier (i.e., of psychopathic traits) to a diagnosis of antisocial personality disorder (ASPD). The psychopathy specifier is regarded as a “distinct variant” of ASPD and is reflective of “primary psychopathy” (APA, 2013, p. 765), which generally refers to interpersonal (as opposed to behavioral) traits commonly associated with psychopathy (e.g., manipulation, superficial charm; Douglas et al., 2015). The specifier is characterized by traits from the FFM domains, namely, Negative Affectivity (i.e., low levels of anxiousness; LLA), Detachment (i.e., low levels of withdrawal; LLW), and Antagonism (i.e., high levels of attention seeking; AS; see Supplemental Table S1), and is conceptualized as a “bold interpersonal style that may mask maladaptive behaviors” (APA, 2013, p. 765). Not surprisingly, the psychopathy specifier has strong conceptual and empirical overlap with the construct of boldness (Anderson et al., 2014; Crego & Widiger, 2015; Wygant et al., 2016). Given the significant controversy over the role of boldness in the conceptualization of psychopathy, the clinical utility of the specifier has been questioned (Crego & Widiger, 2015; Gatner et al., 2016). In keeping with the stated guidelines for revising the *DSM*—that proposed changes should be accompanied by empirical validation and a consensus among experts (Kendler et al., 2009)—empirical research on the construct validity and clinical utility of the psychopathy specifier is warranted.

### Section III ASPD

Research conducted with university, community, and custody samples as well as mental health professional prototypicality ratings have found empirical support for concurrent validity between Section III ASPD traits and other measures of psychopathy (i.e., PCL-R, TriPM; Anderson et al., 2018; Strickland et al., 2013; Wygant et al., 2016, 2020). Furthermore, a study with criminal defendants undergoing evaluation found conceptual support for the psychopathy specifier at the domain level (Wygant & Sellbom, 2012). However, studies that examined concurrent associations between the specifier and measures of psychopathy have found concurrent associations between the specifier and boldness (i.e., EPA, TriPM, PPI-R, and the Self-Report Psychopathy Scale [SRP:III]; Paulhus et al., 2009) but weak or inverse associations with other psychopathy facets and total scores (Anderson et al., 2014; Crego & Widiger, 2014; Few et al., 2015; Miller et al., 2018; Wygant et al., 2020). Moreover, at the specifier facet level, prior investigations have found that one facet (i.e., LLA) had inverse associations with psychopathy (Crego & Widiger, 2014), but another facet (i.e., AS) had positive associations (Miller et al., 2018) with Factor 1 psychopathy (i.e., the primary or interpersonal components of psychopathy), highlighting the multidimensional nature of the specifier. In addition, using a sample of criminal justice–involved women, Wygant and colleagues (2020) found that AS predicted TriPM and SRP-4 (Paulhus et al., 2017) psychopathy.

In addition to its construct validity, there is also some evidence that the psychopathy specifier has incremental predictive validity. Specifically, when examining incremental predictive validity for psychopathy above and beyond ASPD traits, most studies have reported that the psychopathy specifier only added incrementally above and beyond Section III ASPD traits when predicting boldness (as per TriPM and PPI-R; Strickland et al., 2013; Wygant et al., 2016). However, one study found evidence of incremental predictive validity above and beyond Section II ASPD (i.e., the current categorical *DSM-5* diagnosis) in the prediction of interpersonal facets of psychopathy (as per the PCL-R; Wygant et al., 2016). With regard to specifier facets, Few and colleagues (2015) found that one facet (i.e., LLW) added incrementally above and beyond Section III ASPD to predict callous affect; however, *lower* levels of this specifier facet were predictive of psychopathy. Most studies have not found evidence of incremental predictive validity of the specifier for externalizing outcomes related to psychopathy (e.g., antisocial behavior; Dunne et al., 2020; Few et al., 2015). However, Miller and colleagues (2018) found that one facet (AS) was positively associated with aggression and substance use.

Taken together, these empirical findings echo theoretical concerns about overemphasizing the role of boldness in psychopathy. In particular, the inverse associations between the specifier and psychopathy facets (e.g., Anderson et al., 2014) raise potential concerns regarding the construct validity and clinical utility and such findings are contradictory to how the specifier is proposed to function (i.e., higher specifier scores ought to be indicative of increased levels of psychopathy). Despite this, opinion on the utility of the psychopathy specifier remains divided. While some scholars have highlighted the validity evidence of the specifier as an intended proxy for boldness (Strickland et al., 2013; Wygant et al., 2016, 2020), others have cautioned against the use of the specifier, especially in the format of a total score, given the observed divergent associations of the three specifier facets (Miller et al., 2018). Furthermore, Crego and Widiger (2014) voiced concerns regarding the psychopathy specifier due to the use of reverse-keyed scales in two of the three specifier facets. Specifically, they cautioned against inferring the presence of certain psychopathic traits (i.e., boldness) on the basis of low scores on a PID-5 domain (Crego & Widiger, 2014). In other words, the absence of one thing does not infer the presence of another.

## Current Study

The aforementioned studies represent preliminary investigations into the role of the psychopathy specifier in a Section III ASPD diagnosis, but empirical and theoretical gaps remain. First, given that both categorical and dimensional approaches to pathological personality emphasize clinically significant distress or functional life impairment (APA, 2013; Frances & Widiger, 2012) and prior empirical associations between psychopathy and broader life dysfunction beyond criminality (Anestis et al., 2019; Donnellan & Burt, 2016; Gatner et al., 2016; Hanniball et al., 2019; Miller & Lynam, 2012), further investigation into the association between the psychopathy specifier and broad life dysfunction is warranted. Indeed, to our knowledge, only one study (i.e., Miller et al., 2018) has investigated convergent associations of the psychopathy specifier beyond criminality and offending (i.e., general maladaptive outcomes). Second, given previous research findings on the positive association between boldness and adaptive functioning (Dotterer et al., 2017; Gatner et al., 2016; Hanniball et al., 2019; Marcus et al., 2013; Miller & Lynam, 2012), examining the association between the specifier and positive functioning is also warranted. Finally, limited research (i.e., Dunne et al., 2020; Miller et al., 2018) has addressed the psychopathy specifier as a multidimensional construct.

To address these empirical gaps, the current research has three main objectives pertaining to the construct- and criterion-related validity of the psychopathy specifier. We expanded upon prior bivariate research and further examined the multivariate associations of the psychopathy specifier (i.e., Criterion B of the AMPD). Specifically, we examined the concurrent, convergent, and discriminant associations between the specifier and various outcomes using a multivariate analysis of variance procedure—canonical correlation analysis (CCA), which takes into consideration the multifaceted nature of the specifier. Given previous findings (e.g., Miller et al., 2018), we predicted that one facet (i.e., AS) would produce the strongest validity evidence.

## Method

### Procedure

There were two samples that used similar procedures (i.e., university students and community residents with criminal histories). In both, participants read descriptions of available studies and independently signed up for the current study. Participants received a secure link to the online questionnaire via Qualtrics (personality and outcome measures were counterbalanced via an online algorithm). The current study was approved by the university’s ethics board.^2^ Participants in the community sample received a renumeration of US$4.50.

### Sample

#### Sample 1: University Sample

The first sample consisted of 224 undergraduate university students from Western Canada recruited through the university’s online research system as part of course credit for first- and second-year introductory psychology courses. A university sample was deemed sufficient for initial investigation of the current research questions given prior research indicating that personality disorders, including psychopathy, exist on a continuum (Edens et al., 2006; Hart & Cook, 2012). Furthermore, community or university samples are commonly used in self-report psychopathy research (e.g., Coffey et al., 2018; Collison et al., 2021; Drislane et al., 2014; Gatner et al., 2016; Miller et al., 2020; Sellbom et al., 2021; Weiss et al., 2021). The sample comprised mainly self-identified women (77.7%) and the mean age was 19.3 (*SD* = 2.4, range = 18–39) years. Less than half of the sample (43.3%) learned English as their first language and participants self-identified from diverse ethnic groups, including 38.4% Caucasian/European, 23.2% South Asian, 25.4% Chinese, 9.8% Filipino, and 22.2% Other groups (i.e., Southeast Asian, West Asian/Middle Eastern, Korean, Arab, Black/African, Hispanic/Latin American, Indigenous, Japanese). The mean years of education was 13.7 (*SD* = 1.3, range = 12–17+).

#### Sample 2: Community Sample With Prior Criminal Involvement

The second sample consisted of 306 participants located in Canada and the United States recruited online through Amazon’s Mechanical Turk (mTurk). As indicated above, community (and mTurk samples) are commonly utilized in self-report psychopathy research (e.g., Collison et al., 2021; Hanniball et al., 2021a, 2021b; Hyatt et al., 2021; Stanton et al., 2021). In line with recommendations from previous research (e.g., Peer et al., 2014), the following inclusion criteria were used to decrease validity concerns: HIT (i.e., task) approval rate of 95% and above and at least 5,000 prior HIT approvals. In addition, the study included the following inclusion criteria: 25 years of age or older and committed a serious criminal offense/extensive criminality in the prior 5 years.^3^ An older (i.e., aged 25 and up) sample with criminal involvement was selected to assess generalizability and to provide additional insight into the dimensional nature of maladaptive antisocial personality traits. About two thirds of the sample comprised self-identified men (62.4%) and the mean age was 36.4 (*SD* = 9.7, range = 25–69) years. A majority of participants (93.8%) learned English as their first language and self-identified from diverse ethnicity groups, with most identifying as Caucasian/European (82.4%), as well as Black/African American (9.8%) and other groups (16.5%; that is, Hispanic/Latin American, Arab, Chinese, Filipino, Indigenous/Native American, Korean, South Asian, Southeast Asian/East Asian). The mean years of education was 14.9 (*SD* = 1.7, range = below 12–20) and a majority of participants were employed full-time (78.1%). Participants reported engaging in the following criminal offenses in the prior 5 years: drug-related (36.3%), theft and robbery (27.4%), personal attack (13.1%; for example, aggravated assault, domestic assault), fraud (12.4%; for example, forgery), vandalism (8.8%), and sex offenses (2.9%).

### Measures

#### Antisocial Personality Traits

##### Psychopathy

The Triarchic Psychopathy Measure (TriPM; Patrick et al., 2009) is a 58-item self-report measure of psychopathy and includes three domains: boldness, meanness, and disinhibition. The three TriPM domains have good to excellent internal consistency across undergraduate (Cronbach’s α = .82–.88; Poy et al., 2014; Sellbom & Phillips, 2013) and justice-involved samples (α = .89–.90; Sellbom & Phillips, 2013).^4,5^

##### The Psychopathy Specifier

The Personality Inventory for *DSM-5* (PID-5; Krueger et al., 2012) is a 220-item self-report measure of the *DSM-5* Section III maladaptive personality trait domains, including the psychopathy specifier domains: Negative Affectivity, Detachment, and Antagonism (APA, 2013). Internal consistency of the PID-5 ranges from acceptable to excellent in undergraduate and community samples (Cronbach’s α = .70–.96; Hopwood et al., 2012; Krueger et al., 2012).

#### Various Life Outcomes

##### Substance Use

The Drug Abuse Screening Test (DAST; Skinner, 1982) is a 28-item yes/no self-report measure of substance use problems and associated difficulties (e.g., social, legal, and medical). For the purposes of the current study, alcohol use was inserted into the standard instructions to make it explicit that “substance use” included alcohol use. Prior research has reported good to excellent reliability for the DAST across clinical, undergraduate, and community samples (α = .89–.94; Skinner, 1982; Taylor et al., 2008; Yudko et al., 2007).

##### Antisocial Behaviors

Antisocial behavior was measured by the Subtypes of Antisocial Behavior (STAB; Burt & Donnellan, 2009), which is a 32-item self-report measure of minor types of antisocial behavior across three domains: physical aggression, rule-breaking, and social aggression. Internal consistency for physical aggression (α = .84–.91), social aggression (α = .82–.90), and rule-breaking (α = .71–.87) has been fair to excellent across undergraduate, community, and psychiatric outpatient samples (Burt & Donnellan, 2009, 2010).

##### Occupational and Educational Dysfunction

The Interpersonal and Organizational Deviance Scale (IODS; Bennett & Robinson, 2000) is a 28-item self-report measure of deviant workplace behavior (e.g., intentionally worked slower; tried to turn others against someone when angry with them) across two scales: interpersonal and organizational deviance. For the purposes of the current study, instructions and questions were adapted to include dysfunction in educational settings, in addition to the workplace. The IODS has established concurrent and discriminant validity (Bennett & Robinson, 2000; Thau et al., 2009) but fair reliability for the interpersonal scale (α = .76–.78) and poor to excellent reliability for the organizational scale (α = .68–.92; Bennett & Robinson, 2000; Thau et al., 2009).

##### Sleep Disturbance

As an indicator of health, sleep disturbance was measured by the *DSM-5* Level 2 Sleep Disturbance Measure (*DSM*-*SD*; PROMIS Health Organization and PROMIS Cooperative Group, 2012), which is an eight-item self-report measure of sleep quality and disturbances in over the prior week. During field trials with adult patients, the *DSM*-*SD* had excellent test–retest reliability (intraclass correlation coefficient = .78; Narrow et al., 2013).

##### Prosocial Functioning

The Prosocialness Scale for Adults (PSA; Caprara et al., 2005) is a 16-item self-report measure of prosocial behavior (e.g., I try to help others; I easily lend money or other things). The PSA has demonstrated excellent internal consistency across international community and undergraduate samples (α = .91–.94; Caprara et al., 2005, 2008, 2012). The Social Emotional Questionnaire (SEQ; Bramham et al., 2009) is a 30-item self-report measure of social and emotional functioning (e.g., I notice when other people are frightened; I am confident meeting new people) across the following subscales: emotion recognition, empathy, and social conformity. The SEQ has established concurrent validity and fair internal consistency (α = .70) across community samples (Bramham et al., 2009).

##### Community Engagement

A 28-item self-report measure of involvement in volunteer and community service behaviors (e.g., volunteering, donating) was developed for the purposes of the current study.

##### Affect

The Positive and Negative Affect Schedule (PANAS; Watson et al., 1988) is a 20-item self-report measure of mood, consisting of 10 positive affective states and 10 negative affective states.^6^ The PANAS subscales have good to excellent reliability (α = .84–.91) among international community, criminally involved, and outpatient samples (Crawford & Henry, 2004; Serafini et al., 2016; Watson et al., 1988).

##### Life Satisfaction

The Satisfaction with Life Scale (SWLS; Diener et al., 1985) is a five-item life satisfaction self-report measure. The SWLS has established reliability (α = .81–.90) and construct, concurrent, and discriminant validity across international community and clinical samples (Aishvarya et al., 2014; Arrindell et al., 1991; Diener et al., 1985; Gouveia et al., 2009).

##### Coping Style

As an indicator of stress management, coping style was measured by the Coping Inventory for Stressful Situations (CISS; Endler & Parker, 1994), which is a 47-item self-report measure of three types of coping during stressful situations: emotion-oriented (e.g., blame myself for having gotten into this situation), task-oriented (e.g., think about how I have solved similar problems), and avoidance (e.g., go for a walk), in addition to two subtypes of avoidance coping: distraction (e.g., take time off and get away from the situation) and social diversion (e.g., spend time with a special person). The CISS has fair to excellent internal consistency (α = .74–.92), across international clinical, undergraduate, and community samples (Endler et al., 1993; Endler & Parker, 1994; McWilliams et al., 2003).

### Analytic Procedures

Descriptive statistics (i.e., mean, standard deviation, and range) were calculated for all major study variables and are reported for both samples in Supplemental Table S3. Bivariate associations between major study variables (i.e., nonparametric zero-order correlation analyses using Spearman’s ρ)^7^ are also included in Supplemental Tables S4 to S6. As indicated above, CCA was utilized in the current study. CCA is an ideal multivariate analysis for the current data due to the presence of numerous variable sets and potentially overlapping constructs. Moreover, CCA minimizes Type I error and is recommended for personality disorder research due to the clustering of such variables (see Klipfel et al., 2017; Sherry & Henson, 2005). A canonical correlation represents an overall indicator of the association between two sets of variables (e.g., specifier facets and TriPM domains). For instance, CCA determines which facets of the psychopathy specifier contribute to the synthetic predictor variable and which facets of the outcome variable (i.e., TriPM psychopathy) contribute to the synthetic criterion variable, producing an overall association. This process was repeated between the psychopathy specifier facets and (a) various adaptive life outcomes and (b) various maladaptive life outcomes. Wilks’ lambda (λ) was used to determine statistical significance, as it has the broadest applicability (Sherry & Henson, 2005).

CCA has numerous assumptions. First, CCA does not require variables to be normally distributed, but it assumes multivariate normality (i.e., the linear combination of variables), which is difficult to test for. When variables are normally distributed, there is a higher likelihood that multivariate normality is present; however, there is no requirement for normally distributed variables in descriptive analyses (Tabachnick & Fidell, 2007). Notwithstanding, the non-normal distribution of some variables in the current study (i.e., meanness, disinhibition, substance use, emotion recognition, and organizational deviance in the university sample; rule-breaking, interpersonal deviance, community service, and negative affect in the community sample) decreases the chance that multivariate normality is present. Next, CCA assumes linearity and homoscedasticity between variables, which was assessed through visual inspection of bivariate scatterplots. Plots with oval-shaped distributions were deemed to have a linear relationship and plots with equal distributions of points along the fit line were considered to have homoscedasticity. There were only a few variables in each sample with severe assumption violations and these were removed from analyses.^8^ Finally, there were a few significant outliers (i.e., roughly less than 5% of the sample) and all such outliers were removed from CCA analyses. A minimum of 10 cases per independent variable are needed for sufficient power (Tabachnick & Fidell, 2007); thus, the current samples have acceptable power for these analyses.

With regard to the validity of responses, the university sample initially comprised 234 respondents; three were removed due to a high likelihood of repetitive or random responding (i.e., survey protocol completed in less than 15 minutes) and seven were removed because they reported they were not fluent in English, resulting in a final sample of 224 participants. The community sample initially comprised 397 respondents; 48 were removed due to validity concerns (e.g., repeat worker identification numbers) and another 43 were removed due to high likelihood of repetitive or random responding (i.e., survey protocol completed in less than 15 minutes), resulting in a final sample of 306 participants. In addition, a final validity check was conducted via the Triarchic Assessment Procedure for Inconsistent Responding (TAPIR; Mowle et al., 2017). Scores on the TAPIR ranged from 0 to 16 (*M* = 6.76, *SD* = 2.94) in the university sample (which was consistent with means observed in the derivation sample and other undergraduate samples; Mowle et al., 2017) and 0 to 24 (*M* = 7.60, *SD* = 4.08) in the community sample. Using tentative cut-scores of >11 for university samples and >13 for offending samples (see Kelley et al., 2018; Mowle et al., 2017), 13 respondents were identified from the university sample and 28 respondents were identified from the community sample. Some of these participants were also identified as significant outliers and were removed from CCA analyses. Removal of the additional identified participants did not significantly affect observed findings (i.e., *r* values did not differ by more than .03). Thus, the identified participants were retained in the final samples (unless identified as outliers) to strengthen statistical power. It is noted that the TAPIR may have identified a higher proportion of cases if used as an initial validity screening measure (i.e., before participants with significant validity concerns were removed).

## Results

### Concurrent Validity: TriPM Scales

**Figure 1:**
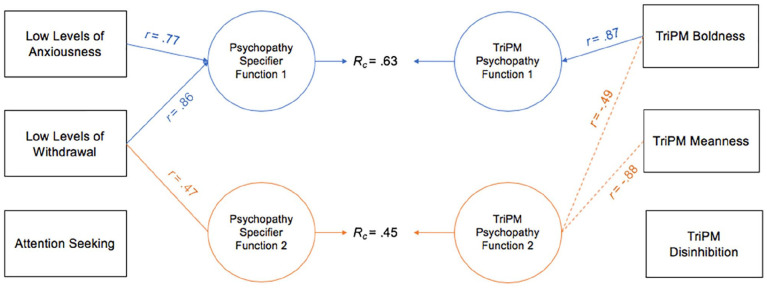
Canonical Solution for Specifier Facets and TriPM Scales: University Sample. *Note.* Table presents first two functions from the canonical solution. *r* = structure coefficient. *R_c_* = canonical correlation between the synthetic predictor and synthetic criterion variables. Only *r* > |.45| are reported. Dotted lines = negative contribution to the synthetic function. TriPM = Triarchic Psychopathy Measure.

**Figure 2.**
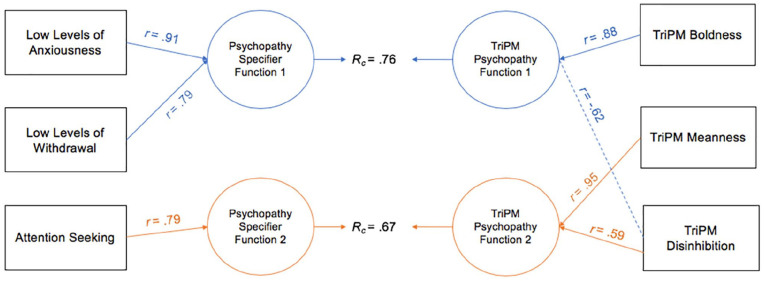
Canonical Solution for Specifier Facets and TriPM Scales: Community Sample *Note.* Table presents first two functions from the canonical solution. *r* = structure coefficient; *R_c_* = canonical correlation between the synthetic predictor and synthetic criterion variables. Only *r* > |.45| are reported. Dotted lines = negative contribution to the synthetic function. TriPM = Triarchic Psychopathy Measure.

CCAs were conducted between the psychopathy specifier PID-5 facets and TriPM scales in both samples (see Tables 1 and 2). Similar to practices in other personality research (e.g., Klipfel et al., 2017), graphical representations of the first two functions (i.e., the interpretable functions) from the canonical solutions between the psychopathy specifier facets and TriPM scales are provided (see Figures 1 and 2).

**Table 1.** Canonical Solution for Specifier Facets and TriPM Scales: University Sample.

Variable	Function 1			Function 2			
	Coef	*r*	*r*^2^ (%)	Coef	*r*	*r*^2^ (%)	*h*^2^ (%)
Boldness	.942	**.865**	74.82	−.305	**−.492**	24.20	**99.02**
Meanness	−.526	−.386	14.90	−.975	**−.883**	77.79	**92.69**
Disinhibition	.049	−.360	12.96	.313	−.033	0.11	13.07
*R_c_*/*R_c_*^2^		.629	39.60		.445	19.82	
Attention seeking	.162	.333	11.09	−.640	−.379	14.36	25.45
Anxiousness (−)	.548	**.770**	59.29	−.757	−.418	17.47	**76.76**
Withdrawal (−)	.612	**.857**	73.44	.929	**.474**	22.47	**95.91**
*F*	23.34***			18.79***			

*Note.* (−) = low levels of the facet (i.e., reverse-scored scales). *N* = 219. *r* > |.45| are in bold. *h*^2^ > 45.00% are in bold. Coef = standardized canonical function coefficient; *r* = structure coefficient; *r*^2^ = squared structure coefficient in the form of a percentage; *h*^2^ = communality coefficient; *R_c_* = canonical correlation between the synthetic predictor and synthetic criterion variables; TriPM = Triarchic Psychopathy Measure.

**Table 2.** Canonical Solution for Specifier Facets and TriPM Scales: Community Sample.

Variable	Function 1			Function 2			
	Coef	*r*	*r*^2^(%)	Coef	*r*	*r*^2^ (%)	*h*^2^ (%)
Boldness	.812	**.880**	77.44	.316	.398	15.84	**93.28**
Meanness	−.076	−.262	6.86	.865	**.954**	91.01	**97.87**
Disinhibition	−.427	−**.621**	38.56	.083	**.586**	34.34	**72.09**
*R_c_/R_c_* ^2^		.756	57.11		.668	44.63	
Attention seeking	.046	.051	2.60	.878	**.790**	62.41	**65.01**
Anxiousness (-)	.702	**.914**	83.54	.523	.163	2.66	**86.20**
Withdrawal (-)	.452	**.789**	62.25	−.663	−.333	11.09	**73.34**
*F*	71.14***			62.14***			

*Note.* (−) = low levels of the facet (i.e., reverse-scored scales). *N* = 303. *r* > |.45| are in bold. *h*^2^ > 45.00% are in bold. Coef = standardized canonical function coefficient; *r* = structure coefficient; *r*^2^ = squared structure coefficient in the form of a percentage; *h*^2^ = communality coefficient;*R_c_* = canonical correlation between the synthetic predictor and synthetic criterion variables; TriPM = Triarchic Psychopathy Measure.

#### University Sample

CCAs conducted between the specifier facets and TriPM psychopathy domains in the university sample produced two interpretable functions with squared canonical correlations of .64 and .45, each contributing to 39.60% and 19.82% of the variance shared between variable sets, respectively.^9^ The full model was statistically significant with a Wilks’ λ of .44, *F*(9, 518.54) = 23.34, *p* < .001, and explained 56.30% of the variance shared between variable sets.^10^ Overall, when examining the communality coefficients (*h*^2^; see Table 1), TriPM boldness and meanness, as well as PID-5 LLA and LLW, all had the highest degrees of usefulness for the CCA solution (i.e., when examining the association between the TriPM domains and PID-5 specifier facets, these variables had the strongest associations with one another); TriPM disinhibition and PID-5 AS did not contribute to this model. When examining Function 1 (i.e., the first representation of the correlation between these two sets of variables), the most relevant TriPM psychopathy criterion variable was boldness, which was evident through the observed large standardized canonical function coefficient (.94) and large canonical structure coefficient (*r* = .87; see Table 1 and Figure 1). In addition, PID-5 LLA and LLW were the most relevant predictor variables (i.e., specifier facets), both having large standardized canonical function coefficients (.55–.61) and large canonical structure coefficients (*r* = .77–.86); however, LLW had a larger contribution to the synthetic predictor variable (73.44% vs. 59.29% for LLA).

Function 2 examines the shared variance left over from Function 1 (i.e., it is the second representation of the correlation between the two sets of variables). In this model, TriPM meanness was the primary criterion contributor, with a large standardized canonical function coefficient (−.98) and large canonical structure coefficient (*r* = −.88; see Figure 1). In addition, TriPM boldness was a secondary contributor, with a moderate standardized canonical function coefficient (−.31) and canonical structure coefficient (*r* = −.49), and PID-5 LLW was the most relevant predictor variable (coef = .93, *r* = .47). In this solution, PID-5 LLW had an inverse association with TriPM meanness (and to a lesser extent TriPM boldness).

#### Community Sample

CCAs conducted on the community sample also produced two interpretable functions with squared canonical correlations of .76 and .67, each contributing to 57.11% and 44.63% of the variance shared between variable sets, respectively.^11^ The full model was statistically significant with a Wilks’ λ of .21, *F*(9, 722.97) = 71.14,*p* < .001, and explained 78.60% of the variance shared between variable sets. All three variables from each set contributed significantly to the CCA solution and were all considered useful in the model (see *h*^2^ values in Table 2). Similar to the university sample, boldness was the strongest TriPM criterion predictor in the first function, which was evident through the observed large standardized canonical function coefficient (.81) and large canonical structure coefficient (*r* = .88). TriPM disinhibition contributed negatively to the criterion variable (coef = −.43, *r* = −.62). In terms of PID-5 specifier facets, LLA was the primary contributor to the synthetic predictor variable and had a large standardized canonical function coefficient (.70) and canonical structure coefficient (*r* = .91), but LLW also had relevant contributions (coef = .45, *r* = .79; see Table 4 and Figure 2). In Function 2, TriPM meanness was the primary criterion contributor (coef = .87, *r* = .95), with a secondary contribution from TriPM disinhibition (coef = .08, *r* = .59).^12^ AS was the only relevant criterion variable, which was evident by a large standardized canonical function coefficient (.88) and large canonical structure coefficient (*r* = .79; see Table 2 and Figure 2).

### Convergent Validity: Maladaptive Life Outcomes

**Figure 3.**
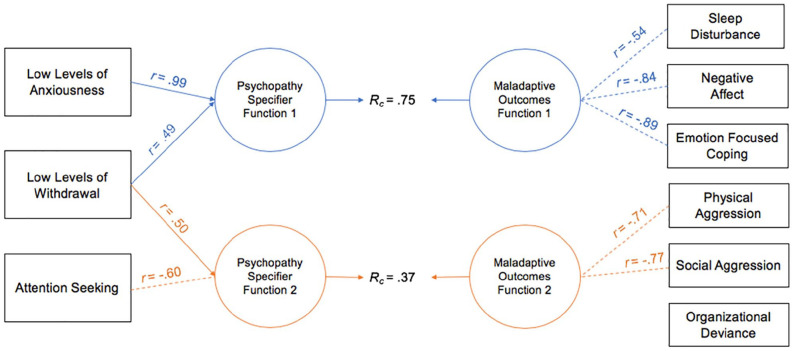
Canonical Solution for Specifier Facets and Maladaptive Life Outcomes: University Sample *Note*. Table presents first two functions from the canonical solution. Only *r* > |.45| are reported. Dotted lines = negative contribution to the synthetic function. *r* = structure coefficient. *R_c_* = canonical correlation between the synthetic predictor and synthetic criterion variables.

**Figure 4.**
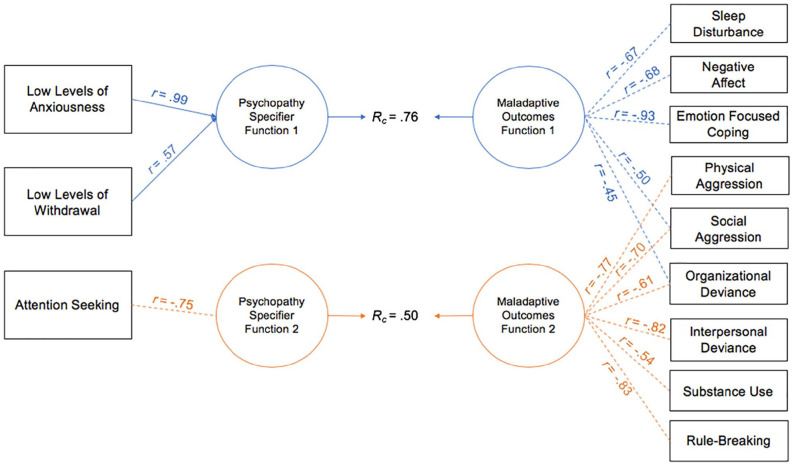
Canonical Solution for Specifier Facets and Maladaptive Life Outcomes: Community Sample *Note*. Table presents first two functions from the canonical solution. Only *r* > |.45| are reported. Dotted lines = negative contribution to the synthetic function. *r* = structure coefficient; *R_c_* = canonical correlation between the synthetic predictor and synthetic criterion variables.

CCAs were conducted between the psychopathy specifier PID-5 facets and various maladaptive life outcomes in both samples (relevant function values are reported in Tables 3 and 4 and graphical representations of the canonical solution are provided in Figures 3 and 4).

**Table 3.** Canonical Solution for Specifier Facets and Maladaptive Life Outcomes: University Sample.

Variable	Function 1			Function 2			
	Coef	*r*	*r*^2^ (%)	Coef	*r*	*r*^2^ (%)	*h*^2^ (%)
Physical aggression	.059	−.195	3.80	−.365	−**.707**	49.98	**53.78**
Social aggression	−.161	−.342	11.70	−.687	−**.766**	58.68	**70.38**
Organizational deviance	.097	−.262	6.86	−.063	−.438	19.18	26.04
Sleep disturbance	−.263	−**.535**	28.62	−.274	−.163	2.66	31.28
Negative affect	−.414	−**.838**	70.22	−.137	−.075	0.56	**70.78**
Emotion focused coping	−.554	−**.892**	79.57	.654	.204	4.16	**83.73**
*R_c_/R_c_* ^2^		.754	56.88		.367	13.52	
Attention seeking	−.069	−.027	0.07	−.830	−**.595**	35.40	35.47
Anxiousness (-)	.923	**.987**	97.42	−.459	−.140	1.96	**99.38**
Withdrawal (-)	.177	.**488**	23.81	.883	**.500**	25.00	**48.81**
*F*	14.31***			6.72***			

*Note.* (−) = low levels of the facet (i.e., reverse-scored scales). *N* = 218. *r* > |.45| are in bold. *h*^2^ > 45.00% are in bold. Coef = standardized canonical function coefficient. *r* = structure coefficient. *r*^2^ = squared structure coefficient in the form of a percentage. *h*^2^ = communality coefficient. *R_c_* = canonical correlation between the synthetic predictor and synthetic criterion variables.

**Table 4.** Canonical Solution for Specifier Facets and Maladaptive Life Outcomes: Community Sample.

	Function 1			Function 2			
Variable	Coef	*r*	*r*^2^ (%)	Coef	*r*	*r*^2^ (%)	*h*^2^ (%)
Substance use	−.089	−.349	12.18	−.108	−**.543**	29.48	41.66
Physical aggression	.084	−.310	9.61	−.286	−**.771**	59.44	**69.05**
Social aggression	−.285	−**.500**	25.00	−.068	−**.702**	49.28	**74.28**
Rule-breaking	.213	−.347	12.04	−.401	−**.830**	68.89	**80.93**
Organizational deviance	−.156	−**.452**	20.43	.216	−**.613**	37.58	**58.01**
Interpersonal deviance	.212	−.270	7.29	−.543	−**.824**	67.90	**75.19**
Sleep disturbance	−.257	−**.669**	44.76	.219	−.026	0.07	44.83
Negative affect	−.158	−**.677**	45.83	−.095	−.364	13.25	**59.08**
Emotion focused coping	−.679	−**.933**	87.05	.285	−.012	0.01	**87.06**
*R_c_/R_c_* ^2^		.759	57.63		.497	24.68	
Attention seeking	−.037	−.069	0.48	−.851	−**.748**	55.95	**56.43**
Anxiousness (-)	.926	**.992**	98.40	−.488	−.093	0.86	**99.26**
Withdrawal (-)	.136	**.570**	32.49	.746	.426	18.15	**50.64**
*F*	15.84***			6.72***			

*Note.* (−) = low levels of the facet (i.e., reverse-scored scales). *N* = 300. *r* > |.45| are in bold. *h*^2^ > 45.00% are in bold. Coef = standardized canonical function coefficient; *r* = structure coefficient; *r*^2^ = squared structure coefficient in the form of a percentage; *h*^2^ = communality coefficient; *R_c_* = canonical correlation between the synthetic predictor and synthetic criterion variables.

#### University Sample

CCAs conducted in the university sample between the PID-5 specifier facets and maladaptive life outcomes produced two statistically significant functions with squared canonical correlations of .75 and .37, each contributing to 56.88% and 13.52% of the variance shared between variable sets, respectively. The full model was statistically significant with a Wilks’ λ of .36, *F*(18, 591.63) = 14.31, *p* < .001, and explained 64.00% of the variance shared between variable sets. Overall, organizational deviance, sleep disturbance, and AS were the only variables that were not deemed useful in the CCA model (see *h*^2^ proportions in Table 3). In Function 1, negative affect and emotion-focused coping were the most relevant criterion variables as evidenced by large canonical structure coefficients (−.84 and −.89, respectively) and large standardized canonical function coefficients (−.41 and −.55, respectively; see Figure 3). Sleep disturbance was a secondary contributor to the synthetic criterion variable (coef = −.26, *r* = −.54). LLA was the only contributing predictor variable to the synthetic predictor as evidenced by a large canonical structure coefficient (.92). However, the predictor and criterion variables were inversely associated (i.e., LLA was inversely associated with emotion-focused coping and negative affect, as well as with sleep disturbance to some extent; see Figure 3).

In Function 2, physical and social aggression were the most relevant criterion variables, as evidenced by large standardized canonical function coefficients (−.37 and −.69, respectively) and large canonical structure coefficients (−.71 and −.77, respectively; see Table 3). In addition, AS and LLW contributed to synthetic predictor variable and had large standardized canonical function coefficients (−.83 and .88, respectively) as well as large canonical structure coefficients (−.60 and .50, respectively); thus, AS had a positive association with these negative outcomes and LLW had an inverse association (see Figure 3). However, this function represents a smaller area of associations among these variables (i.e., only contributed to 13.52% of the variance).

#### Community Sample

CCAs conducted in the community sample between the psychopathy specifier facets and maladaptive life outcomes produced two interpretable functions with squared canonical correlations of .76 and .50, each contributing to 57.63% and 24.68% of the variance shared between variable sets, respectively. The full model contributed to 69.90% of the variance shared between variable sets and was statistically significant with a Wilks’ λ of .30, *F*(27, 841.75) = 15.84, *p* < .001. In Function 1, multiple maladaptive life outcomes contributed to the synthetic criterion variable; emotion-focused coping, negative affect, and sleep disturbance were the most relevant variables, as evidenced by large canonical structure coefficients (−.93, −.68, and −.67, respectively; see Table 4 and Figure 4). Social aggression and organizational deviance also had secondary contributions (coefs = −.29 and −.16, *r* = −.50 and −.45, respectively). In terms of specifier facets, LLA was the primary inverse contributor (coef = .93, *r* = .99), with LLW as a secondary inverse contributor (coef = .14, *r* = .57), albeit moderately. Thus, LLA and LLW had inverse associations with these maladaptive life outcome variables.

Function 2 only contributed to a low percentage of variance between the two variable sets. Rule-breaking, interpersonal workplace deviance, and physical aggression were the strongest contributors (*r* = −.83, −.82, −.77, respectively), followed by social aggression, organizational workplace deviance, and substance use (*r* = −.70, −.61, −.54; see Figure 4). Similar to the university sample, AS was the primary contributor to the synthetic predictor variable with a large standardized canonical function coefficient (−.85) and large canonical structure coefficient (−.75; see Figure 4) and evidenced positive associations with the aforementioned life outcomes.

### Discriminant Validity: Adaptive Life Outcomes

**Figure 5.**
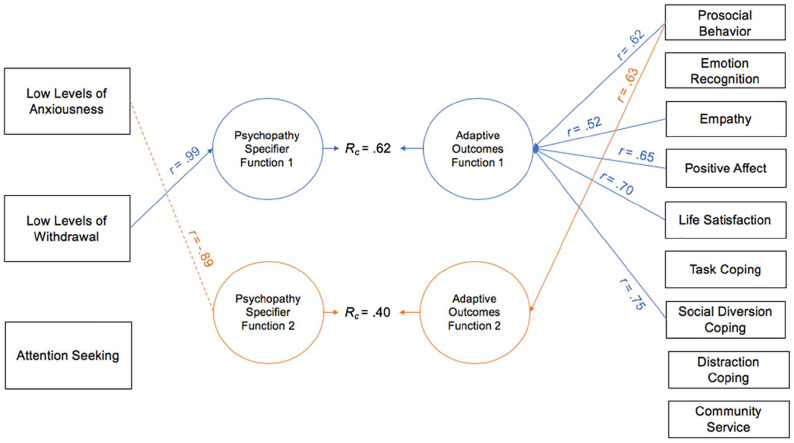
Canonical Solution for Specifier Facets and Adaptive Life Outcomes: University Sample *Note*. Table presents first two functions from the canonical solution. Only *r* > .|45| are reported. Dotted lines = negative contribution to the synthetic function. *r* = structure coefficient; *R_c_* = canonical correlation between the synthetic predictor and synthetic criterion variables.

**Figure 6.**
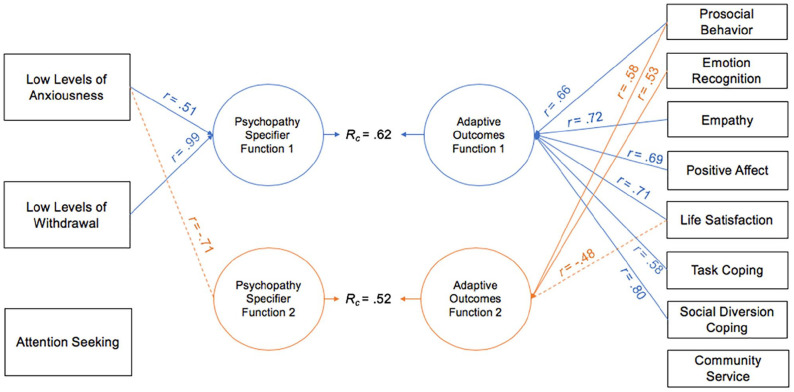
Canonical Solution for Specifier Facets and Adaptive Life Outcomes: Community Sample. *Note*. Table presents first two functions from the canonical solution. Only *r* > .|45| are reported. Dotted lines = negative contribution to the synthetic function. *r* = structure coefficient; *R_c_* = canonical correlation between the synthetic predictor and synthetic criterion variables.

As with maladaptive life outcomes, CCAs were conducted between the psychopathy specifier facets and various adaptive life outcomes in both samples and relevant function values are reported in Tables 5 and 6 and graphical representations are provided in Figures 5 and 6.

**Table 5.** Canonical Solution for Specifier Facets and Adaptive Life Outcomes: University Sample.

Variable	Function 1			Function 2			
	Coef	*r*	*r*^2^ (%)	Coef	*r*	*r*^2^ (%)	*h*^2^ (%)
Prosocial behavior	.171	**.616**	37.95	1.05	**.629**	39.56	**77.51**
Emotion recognition	.019	.405	16.40	.069	.210	4.41	20.81
Empathy	.137	**.519**	26.93	−.278	.217	4.71	31.64
Positive affect	.320	**.646**	41.73	−.351	−.282	7.95	**49.68**
Life satisfaction	.375	**.696**	48.44	−.517	−.413	17.06	**65.50**
Task coping	−.282	.200	4.00	.249	−.010	0.01	4.01
Social diversion coping	.562	**.746**	55.65	.107	.248	6.15	**61.80**
Distraction coping	−.181	−.001	0.00	.166	.229	5.24	5.24
Community service	−.049	.325	10.56	−.223	−.052	0.27	10.83
*R_c_/R_c_* ^2^		.620	38.47		.404	16.35	
Attention seeking	.089	.361	13.03	−.085	.060	0.36	13.39
Anxiousness (−)	.080	.436	19.00	−1.08	−**.893**	79.74	**98.74**
Withdrawal (−)	.938	**.994**	98.80	.506	.071	0.50	**99.30**
*F*	6.529***			3.68***			

*Note.* (−) = low levels of the facet (i.e., reverse-scored scales). *N* = 217. *r* > |.45| are in bold. *h*^2^ > 45.00% are in bold. Coef = standardized canonical function coefficient; *r* = structure coefficient; *r*^2^ = squared structure coefficient in the form of a percentage; *h*^2^ = communality coefficient; *R_c_* = canonical correlation between the synthetic predictor and synthetic criterion variables.

**Table 6.** Canonical Solution for Specifier Facets and Adaptive Life Outcomes: Community Sample.

	Function 1			Function 2			
Variable	Coef	*r*	*r*^2^ (%)	Coef	*r*	*r*^2^ (%)	*h*^2^ (%)
Prosocial behavior	.170	**.660**	43.56	.687	**.582**	33.87	**77.43**
Emotion recognition	−.179	.324	10.50	.329	**.529**	27.98	38.48
Empathy	.327	**.717**	51.41	−.088	.408	16.64	**68.05**
Positive affect	.209	**.687**	47.20	−.471	−.432	18.66	**65.86**
Life satisfaction	.310	**.710**	50.41	−.430	−**.478**	22.84	**73.25**
Task coping	.065	**.581**	33.76	−.103	−.080	0.64	34.40
Social diversion coping	.441	**.797**	63.52	.254	.124	1.54	**65.06**
Community service	−.283	.144	2.07	−.074	−.180	3.24	5.31
*R_c_/R_c_* ^2^		.618	38.22		.520	27.04	
Attention seeking	.074	.176	3.10	−.541	−.432	18.66	21.76
Anxiousness (−)	.035	**.507**	25.71	−1.03	**−.707**	49.98	**75.69**
Withdrawal (−)	.972	**.997**	99.40	.621	.058	0.34	**99.74**
*F*	13.80***			11.23***			

*Note.* (−) = low levels of the facet (i.e., reverse-scored scales). *N* = 303. *r* > |.45| are in bold. *h*^2^ > 45.00% are in bold. Coef = standardized canonical function coefficient; *r* = structure coefficient; *r*^2^ = squared structure coefficient in the form of a percentage; *h*^2^ = communality coefficient; *R_c_* = canonical correlation between the synthetic predictor and synthetic criterion variables.

#### University Sample

CCAs between psychopathy specifier facets and adaptive life outcomes conducted on the university sample produced two interpretable functions with squared canonical correlations of .62 and .40, each contributing to 38.47% and 16.35% of the variance shared between variable sets, respectively. The full model was statistically significant with a Wilks’ λ of .47, *F*(27, 599.35) = 6.53, *p* < .001, and contributed to 52.90% of the variance shared between variable sets. Overall, the following outcome variables contributed significantly to the canonical solution: prosocial behavior, life satisfaction, social diversion coping, positive affect, LLA, and LLW (see *h*^2^ values in Table 5). The most relevant criterion variable in Function 1 was social diversion coping, which had a large canonical structure coefficient (.75), and life satisfaction, positive affect, prosocial behavior, and empathy were also secondary contributors (*r* = .70, .65, .62, and .52, respectively; see Figure 5). The most significant contributor to the synthetic predictor variable was LLW (coef = .94, *r* = .99; see Figure 5). Thus, LLW had positive associations with these adaptive life outcomes. Function 2 contributed to a small amount of variance between the two variable sets and involved only two variables. Specifically, prosocial behavior was the primary contributor among criterion variables (*r* = .63) and LLA was the relevant specifier facet (*r* = −.89) and these variables were inversely associated (see Figure 5).

#### Community Sample

CCAs conducted on the community sample between the psychopathy specifier facets and adaptive life outcomes produced two interpretable functions with squared canonical correlations of .62 and .52, each contributing to 38.22% and 27.04% of the variance shared between variable sets, respectively. The full model was statistically significant with a Wilks’ λ of .38, *F*(24, 847.49) = 13.80, *p* < .001, and contributed to 61.60% of the variance shared between variable sets. Overall, most variables contributed significantly to the model, except for emotion recognition, task-oriented coping, community service, and AS (see *h*^2^ values in Table 6). When examining Function 1, the strongest contributing criterion variables were social diversion coping, empathy, and life satisfaction, which had large canonical structure coefficients (.80, .72, and .71, respectively). Positive affect, prosocial behavior, and task-oriented coping were also secondary contributors (*r* = .69, .66, and .58, respectively). LLW was the strongest predictor variable (coef = .97, *r* = .99), evidencing positive associations with the adaptive life outcomes, and low levels of anxiousness made secondary, albeit smaller, contributions (*r* = .51; see Figure 6). In Function 2, prosocial behavior, emotion recognition, and life satisfaction were primary contributors, though at a moderate level (*r* = .58, .53, and −.48, respectively), and LLA was the primary contributor to the synthetic predictor variable, with a large canonical structure coefficient of −.71. Here, LLA had a positive association with life satisfaction and an inverse association with prosocial behavior and emotion recognition (see Figure 6).

## Discussion

The current study built upon the existing empirical evidence regarding the *DSM-5* Section III alternative model for personality disorder diagnosis specifically related to the diagnosis of ASPD and psychopathy. In the alternative model, a diagnosis of ASPD is more akin to accepted conceptualizations of psychopathy; however, the specifier for the presence of psychopathy has raised concerns among scholars (e.g., see Crego & Widiger, 2014; Few et al., 2015; Miller et al., 2018). Using samples of undergraduate students and community adults with prior criminal involvement and multivariate exploratory analyses, we aimed to replicate prior research and fill empirical gaps pertaining to the construct- and criterion-related validity of the psychopathy specifier by addressing the research questions below.

Overall, as an indicator of psychopathy, we would expect the specifier facets to be associated with a measure of psychopathy (i.e., the TriPM domains). However, our findings indicated that only one specifier facet (i.e., AS) evidenced associations with TriPM meanness and disinhibition. Instead, the remaining facets (i.e., LLA and LLW) only had positive associations with TriPM boldness. In addition, as an indicator of psychopathy, we would expect the specifier facets to have positive associations with areas of life dysfunction commonly observed in psychopathy (e.g., broader antisocial behavior, substance use, negative emotionality). However, we found that two specifier facets (i.e., LLA and LLW) had inverse associations with such outcomes and only one facet (i.e., AS) evidenced positive associations in the manner that one might expect from an indicator of psychopathy. Finally, we would expect that an indicator of psychopathy would generally be unrelated (or inversely related) to adaptive life functioning. However, we observed counterintuitive positive associations between one specifier facet (i.e., LLW) and adaptive life outcomes (e.g., coping, positive behaviors). Another facet (i.e., LLA) had similar counterintuitive associations, in addition to a few inverse associations (i.e., with prosocial behavior and life satisfaction) that we might expect. Moreover, the last facet (i.e., AS) did not have any significant inverse or positive associations with such outcomes.

### What Is the Concurrent Validity of the Psychopathy Specifier?

When examining the association between TriPM psychopathy scales and the psychopathy specifier facets (i.e., identifying which TriPM domains and which specifier facets are associated with one another), two main CCA functions were observed. In the first function, LLA and LLW were positively associated with boldness, with LLA having a stronger association in the community sample. In the second function, meanness was inversely associated with LLW in the university sample and positively associated with AS in the community sample (disinhibition also exhibited this association, but to a lesser extent). Thus, when individuals have increased levels of maladaptive personality traits, AS appears to function in a manner that is related to interpersonal callousness, perhaps tapping into a construct that was intended for the psychopathy specifier. In sum, across two samples, there was weak evidence of concurrent validity of the specifier, as only one facet—AS—in one sample (i.e., the community sample) was associated with TriPM psychopathy (beyond the aforementioned associations with boldness).

### What Is the Convergent Validity of the Psychopathy Specifier?

Exploratory multivariate analyses were conducted to assess whether measures of constructs that ought to theoretically be associated with one another (i.e., psychopathy and life dysfunction) are in fact associated. The first function of CCAs conducted in both samples captured maladaptive life outcomes related to negative emotionality (i.e., negative affect and emotion-focused coping) as being inversely related to LLA. However, in the community sample, significant inverse associations were also observed between LLA (and to some extent, LLW) and more diverse general life dysfunction relating to emotionality, health, dishonesty, and interpersonal conflict (i.e., sleep disturbance, low integrity/poor conduct in the workplace, and socially aggressive behavior). Notably, these findings indicate that as fearlessness and sociability increase, negative behavioral outcomes decrease, suggesting that these facets might actually be protective—contrary to how we might expect an indicator of psychopathy or ASPD to manifest. Similarly, in both samples, the second function captured the positive association between interpersonal aggressiveness and AS, but in the community sample, the positive association was expanded to broader antisocial behavioral outcomes (i.e., rule-breaking, workplace deviance, and substance use). Overall, analyses suggested that fearlessness (i.e., LLA) was the largest (negative) contributor to the association between the psychopathy specifier and maladaptive life outcomes (namely, negative emotionality); thus, LLA was inversely related to these negative life outcomes. In addition, AS was the only facet that had positive associations with maladaptive outcomes (namely, interpersonal and affective life disruption, in addition to externalizing and antisocial behaviors). Therefore, validity evidence of convergent associations between the specifier and maladaptive life outcomes associated with psychopathy was only found in one facet of the specifier—AS, suggesting that the specifier may not capture or relate to important areas of dysfunction commonly observed among those with problematic personality constellations.

### What Is the Discriminant Validity of the Psychopathy Specifier?

Multivariate analyses were conducted to examine whether measures of constructs that ought not be theoretically associated with one another indeed have no association or inverse associations (i.e., criteria anticipated to be inversely related to psychopathy). In the first function, LLW (i.e., sociability) was positively associated with adaptive life indicators related to happiness and social functioning (i.e., social diversion coping and life satisfaction, and to a lesser extent, positive affect, task-focused coping, prosocial behaviors, and empathy). In the second function, both samples captured the inverse association between fearlessness (i.e., LLA) and prosocial behavior (i.e., helping; sharing). In the community sample, fearlessness was inversely associated with emotional functioning, but also positively associated with life satisfaction. Overall, multivariate analyses suggested that LLW had a strong positive association with life satisfaction and healthy social coping strategies, which is counterintuitive for an indicator of psychopathy. On the contrary, LLA was inversely related to prosocial behavior; however, this validity evidence was only observed in one facet of the specifier (and with limited life outcomes). The present study expanded on various outcome variables beyond what has been examined in prior work (e.g., affect, life satisfaction, coping styles, sleep disturbance, and broader prosocial and antisocial behaviors). Interestingly, the strongest inverse associations were observed with sleep disturbance, negative affect, and emotion-focused coping, which are variables that prior research has not yet investigated and are perhaps constructs associated with more general life dysfunction related to personality disorder.

### Implications for Theory, Research, and Clinical Practice

In order for empirical evidence to inform theory, research, and clinical practice, findings ought to be critically synthetized. Specifically, based on existing work on (a) the alternative model of ASPD, (b) the psychopathy specifier, and (c) the construct of boldness, there may be multiple possible explanations for the lack of concurrent, convergent, and discriminant validity for two of three of the PID-5 specifier facets.

First, it is possible that the specifier is capturing what it is intended to, which is a construct that “masks” life dysfunction (e.g., as posited by Patrick, 2018; Strickland et al., 2013), and might be a helpful indicator or consideration in clinical case formulation. However, such a position (e.g., Patrick, 2006, 2018) refers to the *absence* of life dysfunction and negative outcomes that might be more evident in other personality configurations. Yet, the “mask” does not appear to intimate the *presence* of adaptive outcomes (as has been found with two specifier facets in the current and previous studies; Few et al., 2015; Miller et al., 2018). Thus, it appears that the specifier traits are functioning in a manner beyond simply “masking” dysfunction.

Second, it is also possible that existing measures (i.e., the PID-5) are not accurately measuring the intended specifier constructs—an argument that has been raised by others (e.g., Crego & Widiger, 2014). Specifically, the PID-5 may be measuring lack of anxiety and lack of social withdrawal while not actually measuring fearlessness or sociability. Thus, findings might represent evidence of poor construct validity, possibly due to the reliance on reverse-keyed items. Notwithstanding, the specifier was an intended proxy for boldness (Wygant et al., 2016), and thus, given the equivocal research-base on boldness, it is unclear whether the intended construct (whether measured soundly via nonreverse-keyed scales) will hold much clinical utility in terms of identifying those with psychopathic-related deficits.

Finally, it is possible, and our opinion, that the intended specifier construct has limited relevance to psychopathy and limited theoretical and clinically relevance to an ASPD diagnosis, a position that has been expressed by others (e.g., Crego & Widiger, 2014). Moreover, in addition to the findings reported in the current study, supplemental bivariate associations reported in Supplemental Tables S5 and S6 found that the psychopathy specifier evidenced more adaptive correlates (as compared with boldness) and that the construct of Section III ASPD + the specifier was a more adaptive construct than Section III ASPD alone. In view of the consistent findings of poor construct, concurrent, convergent, discriminant, and predictive validity of the specifier across multiple studies (e.g., Crego & Widiger, 2014; Few et al., 2015; Miller et al., 2018), including the current study, it is our view that boldness likely has little relevance to the construct of psychopathy and antisocial personality. Thus, a specifier modeled after this construct likely has little utility in an ASPD diagnosis.

### Limitations

First, the current research examined traits (on a continuum) versus trait extremity and severity (as reflected through disorder-level pathology), and the nature of the samples may limit the external validity of the study (i.e., generalizability to corrections or mental health samples). However, as personality disorders, including psychopathy, are conceptualized to exist on a continuum (Hart & Cook, 2012), and the *DSM-5* alternative model represents maladaptive variants of normal personality traits, university and community samples do not significantly limit the ability to answer the current research questions (e.g., see other studies conducted with such samples: Collison et al., 2021; Drislane et al., 2014; Miller et al., 2020; Weiss et al., 2021). Therefore, if psychopathy represents extreme variants of typical personality, theoretically, similar trends ought to be visible across various samples.

Second, there might be a sampling and selection bias in who chose to sign up for the current online studies, which might affect internal validity. In addition, the groups differed significantly in English as a first language: 43.3% in the university sample versus 93.8% in the community sample, χ^2^(1, *N* = 521) = 187.38, *p* < .001. However, English proficiency was required for university admission, so this was not considered to be a confound. Notwithstanding, the current university sample may differ from some American samples due to the presence of considerable cultural diversity and a large proportion of non-native English speakers. Indeed, this can be considered a strength of the current research—consistent trends were observed across quite different samples.

Third, these findings may also be affected by general issues related to self-report surveys broadly (i.e., self-report bias, inaccuracy, carelessness, and especially as related to psychopathy, dishonesty). However, procedures were implemented to detect this (e.g., survey length was screened and cases with low response times were removed).^12^ Moreover, some research has found that mTurk data have replicated laboratory studies (see Mason & Suri, 2012) and is considered a viable method for conducting personality disorder research (Hauser & Schwarz, 2016; Miller et al., 2017; cf. Gleibs, 2017). Almost *all* research on boldness has used self-report, so our methods in this regard prevent an additional source of variance (method of rating) from confounding observations.

Fourth, the current research used a mono-method investigation into personality constructs wherein personality traits and outcomes were measured at a single time point. Further research may benefit from multimethod investigation (e.g., informant ratings of personality and life outcomes) that also considers the temporal sequencing of the measurement of traits and outcomes. Fifth, we observed that some variables had skewed distributions. Thus, observed null findings for some of these variables may not represent true null findings. In addition, although not observed in a large proportion of our analyses, we observed some violations of the assumptions of linearity and homoscedasticity. Thus, analyses may underestimate the strength of the observed associations or have failed to capture other important associations. Multivariate analyses were also limited in scope due to the potential heteroscedasticity and nonlinearity observed in other study variables that were not included in analyses.

Finally, due to an unanticipated data error, participants in the university sample completed eight items from the AS facet approximately 1 year after the initial data collection. The gap between these personality items and initial data collection could capture differing aspects of personality and life functioning. Notwithstanding, if personality traits are stable (as per common conceptualizations of the development of personality), the potential variability in the measurement of these traits ought to be small (i.e., within-participant variability would not be observable or significant). In addition, the items were measured in isolation, which may raise validity concerns regarding response style; however, the pattern of results associated with AS was similar across samples.

### Future Directions

Future research ought to focus on addressing the potential alternative explanations of existing research findings with an emphasis on construct validity. Specifically, if low neuroticism/high extraversion traits are deemed to be relevant to psychopathy and antisocial personality, then theoretical and empirical work ought to address how and why they are relevant for (a) diagnosis, (b) case formulation, (c) treatment planning, and (d) risk assessment. For instance, would such traits be considered resilience factors in a violence risk assessment? And if so, how do researchers and clinicians make sense of a psychopathy domain that is protective? Future research would also benefit from focusing on trait extremity, severity, and impairment (i.e., through clinical samples as opposed to convenience samples) to make informed conclusions regarding the formalized nosological operationalization of ASPD and psychopathy.

Revisiting the guidelines for revising the *DSM-5* (Kendler et al., 2009), further empirical investigation on professional opinion is warranted, as there appears to be significant disagreement within academic and clinical communities as to the precise nature of psychopathy. It would be beneficial to empirically solicit the opinions of clinical experts on how ASPD/psychopathy should be represented in the next iteration of the *DSM*. Finally, some of the positive associations found between the psychopathy specifier (and boldness) and adaptive outcomes might reflect a tendency among such individuals to be self-promoters and wanting to be seen in a positive light (an idea buttressed by the high correlations with positive impression management and similar scales). Thus, future researchers ought to also focus on observer-rated personality traits and life outcomes to triangulate information and increase the reliability of self-reports. Ideally, future studies would assess trait presence in addition to functional impairment (i.e., Criterion A and B of the AMPD), through self-report, informant-reports, and observer-reports in a clinically relevant sample.

## Conclusion

The current research provided further empirical evidence regarding the multidimensional nature of the psychopathy specifier and examined the construct-and criterion-related validity of the specifier for a diagnosis of ASPD in the *DSM-5* alternative model of personality disorders. Overall, the psychopathy specifier displayed validity evidence similar to that of prior research (e.g., Few et al., 2015; Miller et al., 2018). Specifically, two of the three specifier facets (i.e., LLA and LLW) lacked evidence of concurrent validity with TriPM psychopathy and convergent and discriminant validity with maladaptive and adaptive life outcomes. One facet (i.e., AS) displayed evidence of concurrent, convergent, and discriminant validity. Taken together, our findings suggest that there is no clear evidence of the clinical utility of the psychopathy specifier as a unitary construct and future researchers ought to focus on theoretical and conceptual development. Thus, given the lack of supporting evidence, we do not recommend proceeding with the current version of the psychopathy specifier in an ASPD diagnosis.

## Supplemental Material

sj-docx-1-asm-10.1177_10731911221124344 – Supplemental material for Concurrent, Convergent, and Discriminant Validity of the DSM-5 Section III Psychopathy Specifier Click here for additional data file. Supplemental material, sj-docx-1-asm-10.1177_10731911221124344 for Concurrent, Convergent, and Discriminant Validity of the DSM-5 Section III Psychopathy Specifier by Erin K. Fuller, Dylan T. Gatner and Kevin S. Douglas in Assessment

## Notes

1. In addition to Criterion A and B, individuals must also meet Criterion C (i.e., functional personality impairments that are pervasive across various contexts, such as personal, social, or occupational settings) and Criterion D (i.e., functional personality impairments that are stable and were present during adolescence; APA, 2013).

2. Due to an unanticipated survey error in the university sample (eight items were omitted from the PID-5 in the Qualtrics survey), an ethics amendment was submitted and approved, and participants were re-contacted to complete the missing items (see the “Limitations” section for a full description of the potential implications of this data error).

3. To screen for prior criminality, participants were also asked to report whether they had committed various crimes over the prior 5 years (e.g., fraud, sex offenses, drug-related offenses, robbery, assault, theft), how many times they had committed such offenses, and if they had been arrested.

4. Cronbach’s alpha was interpreted as per Nunnally and Bernstein (1978). Values of .90 and greater are considered excellent, values between .80 and .89 are considered good, values between .70 and .79 are considered fair, and values less than .69 are considered poor.

5. See Supplemental Table S2 for internal consistency values for major study variables in the current study.

6. Two additional items (happy and sad) were added to the scale, as per recent empirical use of the tool (e.g., see Aknin et al., 2013).

7. Due to some normality assumption violations and for consistency in the reporting of results, nonparametric zero-order correlation coefficients (i.e., Spearman’s ρ) were conducted to assess bivariate associations between major study variables.

8. The following variables had severe linearity and homoscedasticity violations in the university sample: substance use, rule-breaking, and interpersonal occupational deviance. In addition, social conformity had linearity and homoscedasticity violations across both samples and distraction coping had violations in the community sample. Thus, given that these analyses are considered exploratory and descriptive, the aforementioned variables were removed from the respective CCA models due to the low likelihood that analyses would be able to detect significant nonlinear associations between the specifier facets and the outcome variables should any associations exist.

9. Only the first two functions were interpreted and reported in Table 3 due to the low squared canonical correlation and low contributed variance of the third and final function, which will be the interpretation pattern for subsequent results.

10. Proportion of variance was calculated from 1 − λ.

11. The community sample had stronger associations between synthetic predictor and criterion variables for each canonical variate function than in the university sample (Function 1: *Z* = −2.769, *p* < .01; Function 2: *Z* = −3.683, *p* < .001). Note it was not meaningful to compare strength of canonical correlations across samples for the life outcome analyses, as different variables were used in each sample.

12. The standardized canonical function coefficient is likely low in this function due to multicollinearity between meanness and disinhibition.
