# Supplementary material for: Concurrent, Convergent, and Discriminant Validity of the DSM-5 Section III Psychopathy Specifier
Source: Assessment. 2022 Sep 19;30(6):1790–810. doi: 10.1177/10731911221124344 (PMC10363948; doi:10.1177/10731911221124344)
Supplement: sj-docx-1-asm-10.1177_10731911221124344 – Supplemental material for Concurrent, Convergent, and Discriminant Validity of the DSM-5 Section III Psychopathy Specifier [file sj-docx-1-asm-10.1177_10731911221124344.docx]

**Table S1**

*Criterion B: Pathological Personality Traits for DSM-5 Section III ASPD Diagnosis*

| ASPD Diagnosis | Psychopathy Specifier |
| --- | --- |
| Disinhibition Domain:   - *Risk Taking Facet* - *Impulsivity Facet* - *Irresponsibility Facet* | Negative Affectivity Domain:   - *Anxiousness Facet (-)*   Detachment Domain:   - *Withdrawal Facet (-)* |
| Antagonism Domain:   - *Manipulativeness Facet* - *Callousness Facet* - *Deceitfulness Facet* - *Hostility Facet* | Antagonism Domain:   - *Attention Seeking Facet* |

*Note.* Must have six or more of the ASPD pathological personality traits for an ASPD diagnosis. (-) = low levels of the facet (i.e., reverse-scored scales).

**Table S2**

*Internal Consistency (Cronbach’s α) of Major Study Variables*

|  | ***Cronbach’s α^a^*** | |
| --- | --- | --- |
|  | **University** | **Community** |
| **Personality Measures**  TriPM Boldness  TriPM Meanness  TriPM Disinhibition  Low Levels of Anxiousness  Low Levels of Withdrawal  Attention Seeking  **Substance Use**  DAST Total  **Antisocial Behavior**  Physical Aggression  Social Aggression  Rule-Breaking  **Occupational Deviance**  Organizational  Interpersonal  **General Health**  Sleep Disturbance  **Prosocial Functioning^b^**  PSA Total  SEQ Emotion Recognition  SEQ Empathy  SEQ Social Conformity  Community Service  **Affect**  Positive Affect  Negative Affect  **Life Satisfaction**  SWLS Total  **Coping Style**  Task Coping  Emotion Coping  Avoidance Coping  Distraction Coping  Social Diversion | .80  .85  .81  .89  .90  .89  .77  .84  .85  .68  .84  .82  .91  .93  .88  .76  .62  .83  .91  .91  .89  .93  .91  .82  .77  .81 | .87  .93  .92  .92  .92  .89  .94  .93  .92  .91  .93  .93  .95  .96  .88  .82  .71  .97  .93  .93  .94  .93  .91  .90  .86  .85 |

*Note*. TriPM = Triarchic Psychopathy Measure. DAST = Drug Abuse Screening Test. PSA = Prosocialness Scale for Adults. SEQ = Social Emotional Questionnaire. SWLS = Satisfaction with Life Scale.

^a^Cronbach’s alpha was interpreted as per Nunnally and Bernstein (1978). Values of .90 and greater are considered excellent, values between .80 and .89 are considered good, values between .70 and .79 are considered fair, and values less than .69 are considered poor. ^b^The SEQ has two additional subscales: sociability and antisocial behavior; however, these subscales had unacceptable reliability (sociability: α = .20, .32; antisocial behavior: α = .09, .22) and were thus, excluded from analyses.

**Table S3**

*Psychometric Properties of Major Study Variables*

|  | **Mean *(SD)*** | | **Range** | |
| --- | --- | --- | --- | --- |
|  | **University** | **Community** | **University** | **Community** |
| **Personality Measures**  TriPM Total  Boldness  Meanness  Disinhibition  Psychopathy Specifier  Anxiousness (-)  Withdrawal (-)  Attention Seeking  **Substance Use**  DAST Total  **Antisocial Behavior**  STAB Total  Physical Aggression  Social Aggression  Rule-Breaking  **Occupational Deviance**  IODS Total  Organizational  Interpersonal  **General Health**  Sleep Disturbance  **Prosocial Functioning**  PSA Total  SEQ Total  Emotion Recognition  Empathy  Social Conformity  Community Service  **Affect**  Positive Affect  Negative Affect  **Life Satisfaction**  SWLS Total  **Coping Style**  Task Coping  Emotion Coping  Avoidance Coping  Distraction Coping  Social Diversion | 54.81 (14.82)  26.79 (8.16)  11.65 (7.60)  16.37 (6.95)  37.99 (12.55)  9.97 (6.49)  18.32 (6.29)  9.70 (5.20)  1.61 (2.30)  54.39 (11.03)  19.07 (5.30)  22.88 (5.63)  12.43 (2.35)  54.42 (20.82)  33.34 (11.84)  14.20 (6.97)  23.59 (7.79)  60.68 (11.34)  110.54 (11.53)  22.22 (3.14)  19.52 (2.95)  12.77 (1.82)  51.04 (13.80)  34.44 (8.39)  26.51 (8.76)  22.71 (6.96)  55.74 (11.72)  49.36 (12.24)  45.47 (10.65)  22.40 (6.41)  15.34 (4.92) | 70.50 (23.90)  29.38 (10.17)  17.34 (12.17)  23.78 (11.97)  37.85 (14.47)  14.52 (7.54)  16.31 (7.70)  7.01 (5.63)  7.37 (5.63)  66.00 (22.57)  20.08 (8.66)  24.40 (7.96)  19.47 (8.09)  70.34 (31.70)  41.61 (18.82)  18.34 (9.94)  21.55 (8.91)  55.52 (15.08)  104.50 (16.74)  19.95 (3.93)  18.02 (4.09)  11.35 (2.60)  53.98 (30.58)  34.48 (10.23)  19.05 (8.63)  19.85 (8.75)  56.85 (12.74)  41.72 (13.60)  44.06 (13.20)  21.59 (7.37)  14.34 (5.13) | 24-129  5-49  0-53  1-45  4-72  0-26  0-30  0-24  0-16  32-96  10-35  11-43  11-24  28-138  16-79  8-43  8-40  22-80  68-138  10-25  6-25  5-15  28-101  12-54  11-55  5-35  16-80  16-75  17-77  8-38  5-25 | 21-150  1-56  0-56  4-55  1-71  0-27  0-30  0-23  0-27  32-154  10-50  11-55  11-49  28-175  16-97  8-56  8-40  16-80  44-141  5-25  5-25  3-15  28-186  12-55  11-50  5-35  20-80  16-77  16-80  8-40  5-25 |

*Note*. (-) = low levels of the facet (i.e., reverse-scored scales). TriPM = Triarchic Psychopathy Measure. DAST = Drug Abuse Screening Test. STAB = Subtypes of Antisocial Behavior. IODS = Interpersonal and Organizational Deviance Scale. PSA = Prosocialness Scale for Adults. SEQ = Social Emotional Questionnaire. SWLS = Satisfaction with Life Scale.

**Table S4**

*Zero-Order Spearman’s Correlations between Psychopathic Specifier and TriPM Psychopathy*

|  | Psychopathy Specifier | Low Levels of Anxiousness | Low Levels of Withdrawal | Attention Seeking |
| --- | --- | --- | --- | --- |
| **University Sample**  TriPM Total  Boldness  Meanness  Disinhibition  **Community Sample**  TriPM Total  Boldness  Meanness  Disinhibition | **.21****  **.57*****  -.13*  -.15*  .13*  **.66*****  -.04  **-.29***** | .12  **.47*****  -.02  **-.28*****  .02  **.59*****  -.05  **-.43***** | -.04  **.36*****  **-.39*****  -.14*  **-.19****  **.47*****  **-.38*****  **-.40***** | **.35*****  **.28*****  .13  .17*  **.58*****  **.29*****  **.48*****  **.41***** |

*Note*. University sample *N* = 218-219. Community sample *N* = 302. Bolded = significant at *p* ≤ .004 after Bonferroni correction applied. * = *p* < .05, ** = *p* < .01, *** = *p* < .001.

**Table S5**

*Zero-Order Spearman’s Correlations for Major Study Variables – University Sample*

|  | B | M | D | TriPM  Total | LLA | LLW | AS | PS | Section III  ASPD | Section III ASPD + PS |
| --- | --- | --- | --- | --- | --- | --- | --- | --- | --- | --- |
| **Substance Use**  DAST Total  **Antisocial Behavior**  STAB Total  Physical Aggression  Social Aggression  Rule-Breaking  **Occupational Deviance**  IODS Total  Organizational  Interpersonal  **General Health**  Sleep Disturbance  **Prosocial Functioning**  PSA Total  SEQ Total  Emotion Recognition  Empathy  Social Conformity Community Service  **Affect**  Positive Affect  Negative Affect  **Life Satisfaction**  SWLS Total  **Coping Style**  Task Coping  Emotion Coping  Avoidance Coping  Distraction  Social Diversion | .04  .05  .06  .02  .01  -.02  -.08  .07  -.13  .08  **.21****  .17*  .13  -.02  **.28*****  **.50*****  **-.39*****  **.34*****  **.33*****  **-.45*****  .15*  -.02  .21** | .10  **.50*****  **.54*****  **.38*****  **.23*****  **.38*****  **.27*****  **.44*****  .03  **-.52*****  **-.58*****  **-.33*****  **-.50*****  **-.48*****  -.15*  -.14*  .15*  **-.29*****  -.19**  -.02  -.00  .11  -.15* | **.26*****  **.44*****  **.34*****  **.39*****  **.37*****  **.42*****  **.38*****  **.39*****  .08  -.11  **-.41*****  **-.30*****  **-.24*****  **-.29*****  -.11  **-.22****  **.33*****  **-.34*****  **-.39*****  **.34*****  **.25*****  **.25*****  .13* | .16*  **.42*****  **.39*****  **.31*****  **.25*****  **.35*****  **.25*****  **.40*****  -.01  **-.23*****  **-.32*****  -.16*  **-.24*****  **-.35*****  .04  .14*  -.02  -.11  -.06  -.12  **.24*****  .17**  .15* | -.13  -.17*  -.11  -.20**  -.07  -.15*  -.16*  -.06  **-.34*****  -.13  .10  -.03  .01  -.06  .07  **.22****  **-.61*****  **.29*****  .00  **-.65*****  .04  -.03  .07 | .02  -.20**  **-.23*****  -.15*  -.08  -.09  -.13  -.01  **-.25*****  **.36*****  **.49*****  .21**  **.29*****  **.22****  .20**  **.34*****  **-.33*****  **.38*****  .10  **-.26*****  **.23****  -.02  **.42***** | .01  .18**  .11  .21**  .03  .18*  .07  **.25*****  .02  .14*  .14*  .10  .05  .02  .18**  **.24*****  .02  .12  .04  -.02  .20**  .15*  .17* | -.07  -.12  -.11  -.11  -.08  -.06  -.12  .05  **-.29*****  .17*  **.39*****  .16*  .20**  .14*  .20**  **.40*****  **-.50*****  **.40*****  .09  **-.50*****  **.21****  .02  **.32***** | .12  **.57*****  **.50*****  **.46*****  **.33*****  **.43*****  **.31*****  **.42*****  .14*  **-.24*****  **-.43*****  -.21**  **-.24*****  **-.28*****  .06  -.05  **.24*****  **-.30*****  -.16*  .19**  .20**  **.26*****  .03 | .09  **.47*****  **.39*****  **.38*****  **.25*****  **.36*****  **.22****  **.42*****  .15*  -.13  **-.23****  -.12  -.15*  **-.23****  .09  .14*  -.01  -.08  -.11  -.05  **.30*****  **.25*****  .20** |

*Note*. DAST = Drug Abuse Screening Test. STAB = Subtypes of Antisocial Behavior. IODS = Interpersonal and Organizational Deviance Scale. PSA = Prosocialness Scale for Adults. SEQ = Social Emotional Questionnaire. SWLS = Satisfaction with Life Scale. B = TriPM boldness. M = TriPM meanness. D = TriPM disinhibition. LLA = low levels of anxiousness. LLW = low levels of withdrawal. AS = attention seeking. PS = psychopathy specifier. Section III ASPD = DSM-5 Section III Antisocial Personality Disorder as per PID-5 domains. Section III ASPD + PS = DSM-5 Section III Antisocial Personality Disorder as per PID-5 domains and the psychopathy specifier PID-5 domains. *Ns* = 199 to 222. Bolded = significant at *p* < .002 after Bonferroni correction applied. * = *p* < .05, ** = *p* < .01, *** = *p* < .001.

**Table S6**

*Zero-Order Spearman’s Correlations for Major Study Variables – Community Sample*

|  | B | M | D | TriPM  Total | LLA | LLW | AS | PS | Section III  ASPD | Section III ASPD + PS |
| --- | --- | --- | --- | --- | --- | --- | --- | --- | --- | --- |
| **Substance Use**  DAST Total  **Antisocial Behavior**  STAB Total  Physical Aggression  Social Aggression  Rule-Breaking  **Occupational Deviance**  IODS Total  Organizational  Interpersonal  **General Health**  Sleep Disturbance  **Prosocial Functioning**  PSA Total  SEQ Total  Emotion Recognition  Empathy  Social Conformity Community Service  **Affect**  Positive Affect  Negative Affect  **Life Satisfaction**  SWLS Total  **Coping Style**  Task Coping  Emotion Coping  Avoidance Coping  Distraction  Social Diversion | -.09  -.10  -.04  -.13*  -.09  -.06  -.12*  .02  **-.36*****  .07  **.20****  .10  .11  -.07  .06  **.50*****  **-.42*****  **.44*****  **.33*****  **-.52*****  .09  -.04  .13* | **.37*****  **.47*****  **.44*****  **.37*****  **.50*****  **.46*****  **.40*****  **.49*****  .14*  **-.65*****  **-.74*****  **-.53*****  **-.64*****  **-.67*****  -.05  -.12*  **.21*****  -.12*  **-.34*****  .16**  -.01  .09  **-.23***** | **.58*****  **.59*****  **.47*****  **.49*****  **.68*****  **.56*****  **.57*****  **.48*****  **.34*****  **-.31*****  **-.52*****  **-.25*****  **-.30*****  **-.30*****  -.05  **-.24*****  **.42*****  **-.30*****  **-.40*****  **.41*****  .11  **.22*****  -.09 | **.45*****  **.48*****  **.44*****  **.36*****  **.54*****  **.48*****  **.42*****  **.49*****  .09  **-.46*****  **-.55*****  **-.36*****  **-.43*****  **-.53*****  -.01  .03  .14**  -.04  **-.24*****  .06  .08  .14*  -.11 | **-.20*****  **-.28*****  **-.19*****  **-.32*****  **-.24*****  **-.28*****  **-.32*****  -.17**  **-.50*****  -.00  **.24*****  -.01  .06  .00  -.02  **.35*****  **-.54*****  **.40*****  **.27*****  **-.70*****  -.04  -.15*  .07 | **-.25*****  **-.39*****  **-.31*****  **-.39*****  **-.37*****  **-.31*****  **-.31*****  **-.25*****  **-.39*****  **.40*****  **.56*****  **.24*****  **.42*****  **.25*****  .17**  **.42*****  **-.39*****  **.42*****  **.34*****  **-.36*****  **.26*****  .03  **.47***** | **.21*****  **.27*****  **.25*****  **.24*****  **.27*****  **.29*****  **.26*****  **.33*****  -.07  -.15**  **-.21*****  **-.19****  -.10  **-.30*****  .17**  .17**  .13*  .12*  -.09  .08  **.31*****  **.23*****  **.21***** | **-.18****  **-.26*****  **-.18****  **-.30*****  **-.24*****  **-.22*****  **-.25*****  -.12*  **-.51*****  .17**  **.35*****  .04  **.23*****  .05  .13*  **.46*****  **-.45*****  **.50*****  **.30*****  **-.52*****  **.25*****  .03  **.38***** | **.49*****  **.63*****  **.53*****  **.49*****  **.61*****  **.57*****  **.54*****  **.54*****  **.26*****  **-.46*****  **-.64*****  **-.35*****  **-.44*****  **-.57*****  -.04  -.17**  **.37*****  **-.23*****  **-.39*****  **.31*****  .07  .16**  -.16** | **.42*****  **.47*****  **.44*****  **.36*****  **.51*****  **.46*****  **.43*****  **.48*****  .08  **-.38*****  **-.48*****  **-.32*****  **-.34*****  **-.54*****  .03  .02  **.18*****  -.05  **-.27*****  .10  .15**  .17**  -.02 |

*Note*. DAST = Drug Abuse Screening Test. STAB = Subtypes of Antisocial Behavior. IODS = Interpersonal and Organizational Deviance Scale. PSA = Prosocialness Scale for Adults. SEQ = Social Emotional Questionnaire. SWLS = Satisfaction with Life Scale. B = TriPM boldness. M = TriPM meanness. D = TriPM disinhibition. LLA = low levels of anxiousness. LLW = low levels of withdrawal. AS = attention seeking. PS = psychopathy specifier. Section III ASPD = DSM-5 Section III Antisocial Personality Disorder as per PID-5 domains. Section III ASPD + PS = DSM-5 Section III Antisocial Personality Disorder as per PID-5 domains and the psychopathy specifier PID-5 domains. *Ns* = 293 to 300. Bolded = significant at *p* < .002 after Bonferroni correction applied. * = *p* < .05, ** = *p* < .01, *** = *p* < .001.
